# Defining an Optimal Sample Size for Corneal Epithelial Immune Cell Analysis Using *in vivo* Confocal Microscopy Images

**DOI:** 10.3389/fmed.2022.848776

**Published:** 2022-06-01

**Authors:** Xin Yuan Zhang, Mengliang Wu, Holly R. Chinnery, Laura E. Downie

**Affiliations:** Department of Optometry and Vision Sciences, University of Melbourne, Parkville, VIC, Australia

**Keywords:** cornea, immune cell, confocal, IVCM, dendritic cell, image, sample size, combinations

## Abstract

**Purpose:**

*In vivo* confocal microscopy (IVCM) images are frequently used to quantify corneal epithelial immune cell (IC) density in clinical studies. There is currently limited evidence to inform the selection of a representative image sample size to yield a reliable IC density estimate, and arbitrary numbers of images are often used. The primary aim of this study was to determine the number of randomly selected, unique IVCM images required to achieve an acceptable level of accuracy when quantifying epithelial IC density, in both the central and peripheral cornea. The secondary aim was to evaluate the consistency and precision of an image selection approach where corneal epithelial IC density was quantified from “three representative images” selected independently by three experienced observers.

**Methods:**

All combinations of two to 15 non-overlapping IVCM images were used for deriving IC density estimates, for both the central and peripheral cornea, in 20 healthy participants; the density value from averaging quantifications in the 16 images was defined as the “true mean”. IC density estimates were compared with the true mean in each corneal region using a mean ratio. Intraclass correlation coefficients (ICCs) were used to evaluate the consistency of the mean ratios of IC density estimates derived from the method involving the manual selection of “three representative images” by the observers. The precision of the IC density estimates was compared to a scenario involving three randomly selected images.

**Results:**

A total of 12 randomly selected, non-overlapping IVCM images were found to be required to produce a corneal epithelial IC density estimate that was within 30% of the true mean, 95% of the time, for the central cornea; seven such images produced an equivalent level of precision in the peripheral cornea. Mean ratios of corneal IC density estimates derived from “three representative images” methods had poor consistency between observers (ICC estimates <0.5) and similar levels of precision when compared with using three randomly selected images (*p* > 0.05 for all comparisons), in both the central and peripheral cornea.

**Conclusions:**

Data presented in this study can inform image selection methods, and the sample size required for a preferred level of accuracy, when quantifying IC densities in the central and peripheral corneal epithelium using IVCM images.

## Introduction

*In vivo* confocal microscopy (IVCM) is a high-resolution tool for non-invasively capturing images of the cornea in living humans. Corneal immune cells (ICs) can be visualized in IVCM images as bright, typically dendriform bodies at the level of the basal epithelium (1). These corneal epithelial ICs are generally considered to represent resident dendritic cells (2), which are involved in immune surveillance, initiating adaptive immune responses (3) and maintaining tissue homeostasis (4). The quantification of epithelial IC density from IVCM images is frequently performed in clinical studies, as a means for considering corneal immune status, particularly in the context of disease (5–12). For example, central corneal epithelial IC density has been described to increase in corneal infections (13), contact lens wear (14) and dry eye disease (15).

A single IVCM image has a relatively small field-of-view, typically 400 μm × 400 μm (i.e., 0.16 mm^2^), equating to approximately 0.2% of the entire corneal area. Due to this limited capture area, it is generally recognized that more than one IVCM image needs to be acquired and analyzed to derive a representative estimate of the corneal epithelial IC density in a particular corneal region. However, there has not yet been a study investigating the optimal IVCM image sample size required to derive a valid estimate of corneal epithelial IC density for a particular individual. Kheirkhah et al. proposed that averaging findings from “three representative images, chosen by an experienced observer,” could accurately estimate central corneal epithelial IC density in a clinical population (16). However, a human observer, experienced or otherwise, may have unconscious biases in image selection, particularly if they are not masked to a participant’s health status; such biases could affect the validity of the epithelial IC density measures (17). Another approach has been to analyze IVCM images with the highest IC density (8, 18), which is likely also problematic as it could overestimate absolute values and/or the effect of an inflammatory overlay.

Previous corneal IVCM studies have used a variety of sample sizes, including quantifications from three (9, 15, 19–22), five (6), eight (23), or twelve (24) non-overlapping images, to derive an estimate of central corneal epithelial IC density for a single participant. Using a larger sample would be expected to yield a more accurate estimate, as a larger portion of the corneal region is directly quantified; the trade-off is that more time and effort is required for the image acquisition, selection and analysis (25). Using randomly selected images, rather than images selected by an observer, would be expected to reduce biases in image selection. However, the optimal image sample size for quantifying corneal epithelial IC density from randomly selected IVCM images is yet to be determined. The effect of corneal eccentricity on the required sample size also requires consideration. Corneal epithelial IC density is eccentricity dependent, with approximately threefold more cells in the peripheral cornea relative to the central region (26). Therefore, different image sample sizes might be required for reliable estimations of central and peripheral corneal IC densities.

Vagenas et al. described a method for determining the optimal sample size of IVCM images for quantifying central corneal sub-basal nerve parameters (27). This study concluded that eight randomly chosen images, overlapping by less than 20%, were needed per participant to produce an estimated value within 30% of the true mean, 95% of the time. The aim of the present study was to use a similar approach to determine the optimal image sample sizes for quantifying epithelial IC density from IVCM images in healthy individuals, for both the central and peripheral cornea. This study also considered whether quantifying the mean number of cells using “three representative images,” by different observers, led to a different corneal epithelial IC density estimate relative to the derived true density.

## Materials and Methods

### Participants

This retrospective study involved the analysis of corneal IVCM images, acquired at the level of the basal epithelium, from 20 randomly selected, heathy adult participants who had participated in research studies in the Downie laboratory at the University of Melbourne from 2017 to 2019. The studies were approved by the University of Melbourne Human Research Ethics Committees (ID #1749830 and #1749836). All participants provided written informed consent to participate. The number of participants was chosen to align with the analysis set defined by Vagenas et al. (27), which adopted a similar methodological approach.

Eligible participants had self-reported no underlying health conditions that could affect eye health (including dry eye disease), were not pregnant or breastfeeding, had not undergone ocular surgery within the 6 months prior to the study visit, and did not have a history of contact lens wear. Dry eye disease symptom screening was conducted using the McMonnies dry eye questionnaire (28); potential participants with a score exceeding 14.5 were ineligible to participate.

### Corneal *in vivo* Confocal Microscopy Image Acquisition and Selection

Participants underwent laser-scanning IVCM (Heidelberg Retina Tomograph-3 with the Rostock Corneal Module, Heidelberg Engineering, Germany) using our established protocols (29). IVCM images (400μm × 400μm) were acquired from the right corneal apex (central region) and 2 mm above the inferior limbus (peripheral region) using the device sequence scan mode, at the level of the basal epithelium. Capturing the relevant regions of interest was achieved by having the contralateral (left) eye focus on a series of fixation targets, involving a grid to ensure the capture of multiple non-overlapping IVCM regions. In total, at least 600 IVCM images were captured per participant, from which 16 unique corneal images from each of the central and peripheral cornea were randomly selected for inclusion in this study. Images that had variable focus, imaging artifacts, compression lines or vignetting effects, and images that captured the same or overlapping corneal regions, were excluded from the analysis set. A total of 640 unique high quality IVCM images comprised the analysis set. This analysis set involved 16 images of corneal areas (defined by <20% overlap with any other image in the analysis set) in both the central and peripheral cornea for each participant. To confirm that images were not overlapping, they were processed using the Photomerge function in Photoshop (Adobe Photoshop Version: 23.0.0) with images that were unable to merge regarded as non-overlapping.

### Image Analysis

**FIGURE 1:**
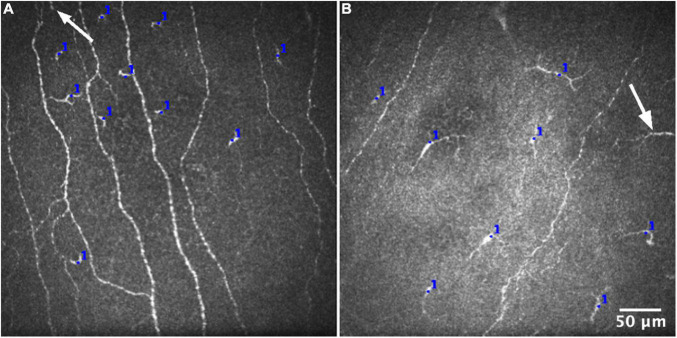
Representative IVCM images for quantifying epithelial immune cell density from the central **(A)** and peripheral **(B)** corneal regions. The number of cells was manually counted in each IVCM image, using the Cell Counter plugin in ImageJ (blue marks). Cells that were only partially visible at the edge of an image (white arrows) were consistently excluded from the analysis.

For each IVCM image, the number of corneal epithelial ICs was manually counted by one experienced observer using the Cell Counter plugin in ImageJ (30) (Figure 1). ICs that were only partially visible at the edge of an image were excluded. Corneal IC density (cells/mm^2^) was calculated for each image. For each participant, the average epithelial IC density from the 16 images (quantified corneal area: 2.56 mm^2^), in both the central and peripheral cornea, was regarded as the reference standard and “true” mean value based on the method used by Vagenas et al. (27).

To evaluate whether this experienced observer’s IC counts were representative of other observers, two other experienced observers also independently performed IC counts in 100 randomly selected IVCM images, comprising 50 from the central cornea and 50 from the peripheral cornea; this subset represents one sixth of the total number of images analyzed for the study. The level of inter-observer agreement was analyzed using the intraclass correlation coefficient (ICC), with 95% confidence intervals, using a single-rating, absolute-agreement, two-way random-effects model.

The analysis also considered how the estimate of corneal epithelial IC density from “three representative images,” selected manually by observers, compared with the estimate derived from random image selection. Three experienced observers were instructed to select three IVCM images, from both the central and peripheral cornea, that they considered to “best represent” the 16 images for each participant. The epithelial IC density estimate for each participant, in each corneal region, was then calculated by averaging the IC densities from the “three representative images” selected by each of the three observers.

### Statistical Analysis

The statistical approach for the current study was based on the methodology described by Vagenas et al. (27). The sampling technique involved creating mathematical combinations of IVCM image sets, comprising two to 15 unique images (*k*) from the 16 image reference set (*n*), in both the central and peripheral regions, for each participant. A combination was defined as an unordered selection of *k* items, from a set of *n* items without repetition (*k* ≤ *n*). Estimates of corneal IC density, determined by sampling different numbers of randomly selected images, were then compared to the “true” mean value in order to determine the minimum number of images that would provide an acceptable estimate, defined as less than 30% different to the “true mean” value; this level of precision was defined as acceptable based on the criterion used for corneal nerve parameter estimates by Vagenas et al. (27). The estimated IC density is presented as the “relative mean,” also termed the “mean ratio,” defined as the ratio between the estimated and “true” IC density, for each participant. Mean ratios were plotted relative to the number of images (two to 15) used to derive the estimates. The same process was followed for both the central and peripheral corneal regions.

Given the large number of estimates generated from each combination level, the mean ratios were considered to approximate a normal distribution, based on the Central Limit Theorem. The confidence intervals (CIs) of the mean ratio data were calculated using the formula CI=μ±(t×*SD*) at confidence levels of 80, 85, 90, and 95% (where μ is the mean of the distribution, SD is the standard deviation of the distribution, and t is the value that corresponds to 80, 85, 90, and 95% levels of confidence in a t-distribution).

The level of consistency between estimates of corneal epithelial IC density derived from the “three representative images” selected by three independent observers was analyzed separately for the central and peripheral regions using the ICC. ICC estimates and their 95% CIs were calculated using a single-rating, consistency-agreement, two-way random-effects model. The three observers were selected randomly and therefore the results are expected to be generalizable to the whole observer population. The difference in mean estimates of IC density ratio between each observer and a random combination of three IVCM images was analyzed using a Student’s *t*-test, with Bonferroni adjustment for multiple comparisons. To evaluate whether the 95% CI estimations for corneal IC density from the observers were narrower (i.e., had better precision) than the 95% CIs derived from a random combination of three IVCM images, variance equality was evaluated using an *F*-test, with Bonferroni adjustment for multiple comparisons.

All statistical analyses were performed, and figures constructed, using R software (Version 4.1.2, R Development Core Team).^1^ A *p*-value less than 0.05 was considered statistically significant.

## Results

### Corneal Immune Cell Density

Overall, the mean (± SD) epithelial IC density for the 20 healthy participants, calculated from the “true” mean quantified from 16 images by a single expert observer for each participant was 21.7 ± 17.7 cells/mm^2^ (range: 2.7 to 63.3 cells/mm^2^) for the central cornea, and 62.0 ± 26.1 cells/mm^2^ (range: 17.2 to 104.7 cells/mm^2^) for the peripheral cornea.

Confirming the validity of the single expert observer’s epithelial IC counts, the ICCs for counts performed independently by three observers in a subset of 100 randomly selected IVCM images was: central cornea: 0.91 (95% CI: 0.85 to 0.95), and peripheral cornea: 0.90 (95% CI: 0.83 to 0.94). These ICCs indicate a high level of agreement in the quantification of corneal epithelial ICs from IVCM images by three experienced observers.

### Optimal *in vivo* Confocal Microscopy Image Sample Size for Estimating Corneal Epithelial Immune Cell Density

**FIGURE 2:**
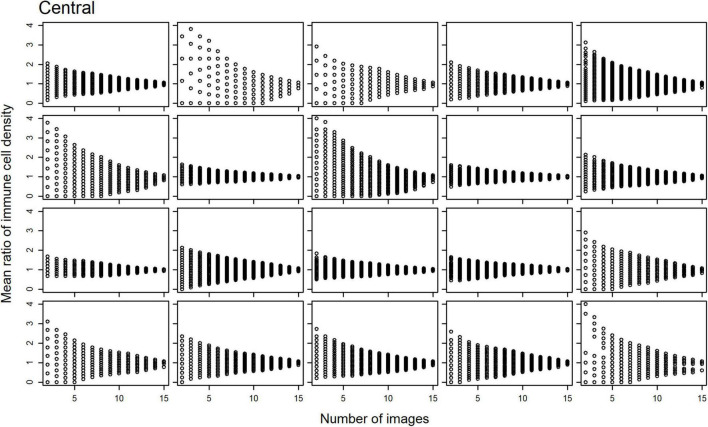
Scatterplots of the mean ratio for corneal epithelial immune cell (IC) density relative to the number of randomly selected, non-overlapping IVCM images used for the density estimate for each participant, in the central cornea.

**FIGURE 3:**
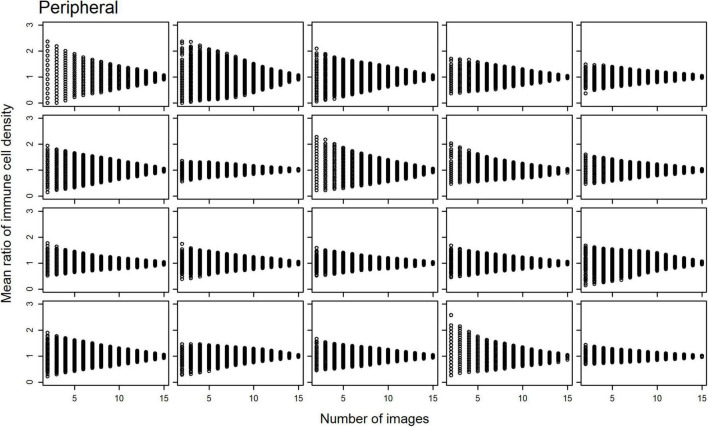
Scatterplots of the mean ratio for corneal epithelial immune cell (IC) density relative to the number of randomly selected, non-overlapping IVCM images used for the density estimate for each participant, in the peripheral cornea.

Scatterplots of the mean ratios for IC density estimates derived from all image combinations, for all study participants, are shown for the central (Figure 2) and peripheral (Figure 3) cornea; as the number of sampled images increases, the spread of the mean ratio data decreases.

**FIGURE 4:**
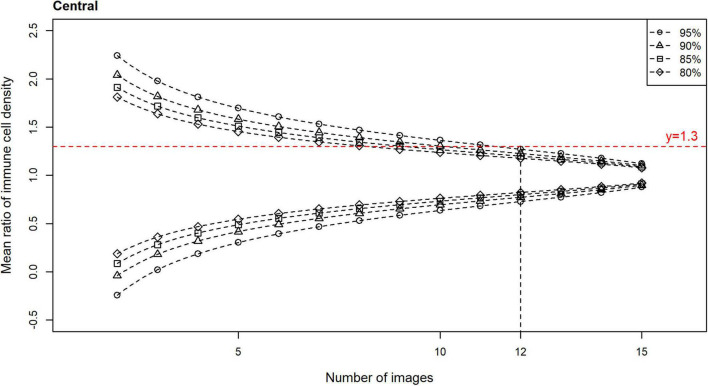
Confidence intervals (CIs) with 80, 85, 90, and 95% levels of confidence for the mean ratio for corneal epithelial immune cell (IC) density relative to the number of randomly selected, non-overlapping images used for the density estimate, for all participants for the central cornea. Horizontal line (mean ratio = 1.3) indicates the location where the estimated mean is 30% different from the true mean in the distributions. Vertical line (number of images = 12) indicates the number of images that is predicted to produce estimated means that are 30% different from the true mean (i.e., mean ratio = 1.3 or 0.7), at a confidence level of 95%, for the central cornea.

**FIGURE 5:**
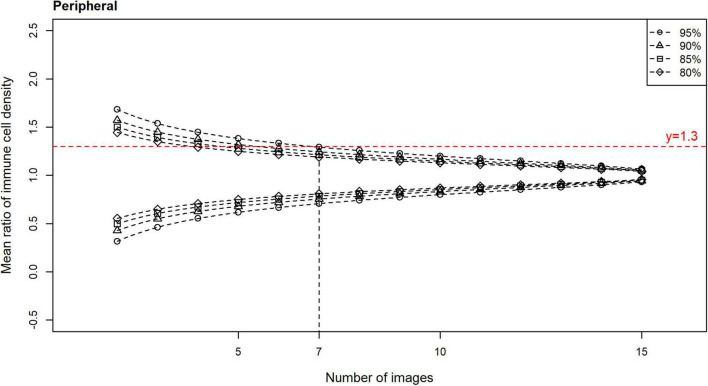
Confidence intervals (CIs) with 80, 85, 90, and 95% levels of confidence for the mean ratio for corneal epithelial immune cell (IC) density relative to the number of randomly selected, non-overlapping images used for IC density estimate, for all participants for the peripheral cornea. Horizontal line (mean ratio = 1.3) indicates the location where the estimated mean is 30% different from the true mean in the distributions. Vertical line (number of images = 7) indicates the number of images that is predicted to produce estimated means that are 30% different from the true mean (i.e., mean ratio = 1.3 or 0.7), at a confidence level of 95%, for the peripheral cornea.

Plots of the CIs (80, 85, 90, and 95%) for the epithelial IC density mean ratios, relative to the number of images used to derive the estimate, are shown for the central (Figure 4) and peripheral (Figure 5) cornea. In both regions, the CIs narrow as the number of images used to derive the estimate increases. An optimal IVCM image sample size to estimate the true corneal epithelial IC density was determined from the plots, using a pre-specified level of precision (mean ratio) and level of confidence. The estimated mean was considered acceptable if it was not more than 30% different from the true mean at a 95% confidence level (27). Using this criterion, 12 randomly selected, non-overlapping images of the central cornea, and seven such images of the peripheral cornea, were found to be required, per participant, for accurate quantification and averaging.

### Evaluation of the Method Involving Selection of the “Three Representative” *in vivo* Confocal Microscopy Images

The ICC for the mean ratio of the epithelial IC density estimates derived from the three images selected as “representative” by three independent observers was −0.21 (95% CI: −0.35 to 0.04) for the central cornea, and 0.17 (95% CI: −0.08 to 0.48) for the peripheral cornea. Given that poor reliability is defined by ICC values less than 0.50 (31), this result indicates poor consistency among the mean ratio estimates of IC density between the three observers.

**FIGURE 6:**
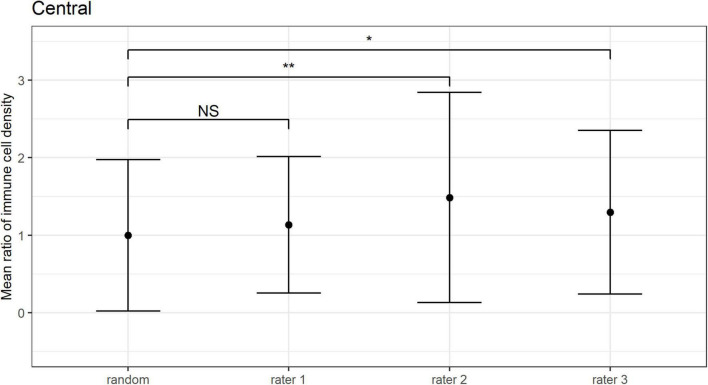
Central corneal epithelial immune cell (IC) density mean ratios, and corresponding 95% CIs, obtained when IC density estimates derived from all combinations of three randomly sampled images (random), and when the “three representative images” were independently selected by three observers (i.e., raters 1 to 3). Mean ratios of IC density estimates derived from images selected by rater 2 and rater 3 were significantly higher in comparison to 3 randomly sampled images (**p = 0.01; *p < 0.05; NS: *p* > 0.05).

**FIGURE 7:**
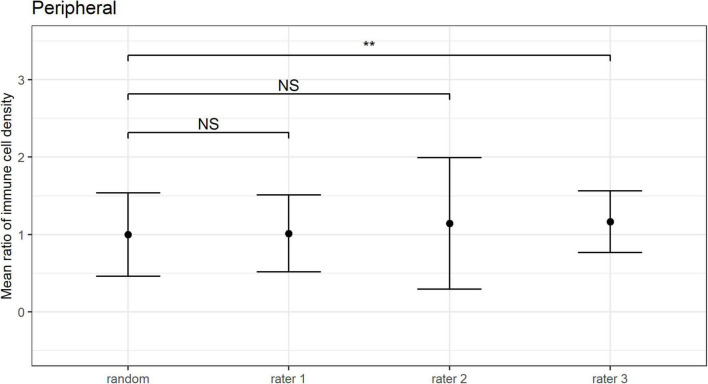
Peripheral corneal epithelial immune cell (IC) density mean ratios, and corresponding 95% CIs, obtained when IC density estimates derived from all combinations of three randomly sampled images (random), and when the “three representative images” were independently selected by three observers (i.e., raters 1 to 3). Mean ratios of IC density estimates derived from images selected by rater 3 were significantly higher in comparison to 3 randomly selected images (**p < 0.01; NS: *p* > 0.05).

The mean ratio and corresponding 95% CIs for epithelial IC density estimates derived from the “representative” images selected by each observer are shown for the central (Figure 6) and peripheral (Figure 7) cornea. Comparing the mean ratio estimates calculated from all combinations of three randomly sampled images, two of the three observers selected images that significantly overestimated central corneal epithelial IC density (rater 2: *p* = 0.010; rater 3: *p* = 0.047), while one observer selected images that overestimated the peripheral corneal IC density (rater 3: *p* = 0.003). The size of the 95% CIs around the epithelial IC density mean ratio for any of the raters was not significantly narrower than the estimate derived from three randomly selected images, in both the central and peripheral cornea (*p* > 0.05 for all comparisons). This finding indicates that there was similar precision in the IC density estimate when three images were subjectively selected by observers and when any random three images were used in the analysis.

## Discussion

This is the first study to investigate the optimal number of IVCM images required to accurately estimate IC density in a healthy individual, in both the central and peripheral corneal epithelium. The analysis also considered the validity of using “three representative images” selected by experienced observers to derive corneal epithelial IC density estimates. The main finding was that to derive a corneal epithelial IC density estimate that is at most 30% different from the “true mean,” 95% of the time, quantifications need to be performed and averaged for 12 randomly selected, non-overlapping IVCM images in the central cornea, and for seven such images in the peripheral cornea, per participant. The study also identified that corneal IC density estimates derived from cell quantifications in “three representative images,” by experienced observers, had poor reliability; overall, the level of precision was similar to using three random images. These findings can inform future IVCM studies that include corneal epithelial IC density calculations, both with respect to the required image sample size and the methods used for image selection.

Quantifying corneal epithelial IC density from IVCM images is frequently used to evaluate corneal inflammation in clinical studies (5–12). Although it is generally accepted that inflamed corneas have higher epithelial IC densities relative to control (healthy) conditions, a recent meta-analysis reported high levels of heterogeneity (*I*^2^ value: 94.5% for the central cornea and 96.1% for the peripheral cornea) among studies that had quantified corneal epithelial IC density from IVCM images in healthy eyes (26). In this analysis, and similar to the data in the present study, the pooled estimate for central corneal epithelial IC density was 26.4 ± 13.6 cells/mm^2^ (from 1203 participants in 38 studies) for the central cornea and 74.9 ± 22.7 cells/mm^2^ (from 466 participants in 9 studies) for the peripheral inferior cornea. The study by Mobeen et al. also investigated whether specific factors, including participant sex, the definition of ICs and whether three or five IVCM images were sampled, contributed to the observed heterogeneity. Age was reported to be the only significant factor, with peripheral corneal epithelial IC density decreasing with advancing age (26). The lack of significance of sample size as a contributing factor in this analysis likely reflects the dichotomous consideration of this variable, and that both categories are relatively under-sampled based on the findings in the present study. Other aspects of the IVCM image analysis approach, such as selection method (e.g., random or observer-selected) may also contribute to the unexplained heterogeneity.

The selection of a subset of images for analysis, from a larger raw acquisition set, is a necessary initial step in IVCM studies. This step has not been consistently performed in previous studies, both in terms of the number of images selected or the method of selection. First, in terms of sample size, it is important to consider whether the number of unique images (each covering 0.16 mm^2^ of corneal area) selected for analysis sufficiently represents the corneal region; under-sampling may lead to inaccurate estimates. The present study identified that to ensure a level of precision such that an estimate was no more than 30% different from the true mean, 95% of the time, at least 12 randomly selected, non-overlapping IVCM images should be used to quantify epithelial IC densities in the central cornea of an individual. Consistent with epithelial ICs being more populous in the peripheral cornea, seven such images were found to be required to achieve the same level of precision in this region. In the absence of evidence to inform optimal image sampling methods, prior clinical studies have used a wide variety of image sample sizes, ranging from three (9, 15, 19–22) to twelve (24) non-overlapping images. The analyses in the current study indicate that central corneal epithelial IC density estimates derived from eight randomly sampled images only reach the above accepted level of precision 80% of the time; with the use of five randomly selected images, the IC density estimates are predicted to only achieve the precision level of not being more than 50% different from the true mean, 80% of the time. Together, these findings suggest studies using less than the determined minimum image sample sizes are at risk of unreliable estimates of corneal epithelial IC density, and report findings should be interpreted in view of this limitation.

Considerations relating to IVCM image sample sizes have also been investigated by Vagenas et al. for quantifying central corneal nerve parameters. These authors concluded that averaging data from at least eight unique IVCM images, per participant, was required to yield an estimate with the same level of precision used in the present study (27). That a larger sample size is required for corneal IC density estimates, relative to nerve density parameters, likely reflects that the healthy central corneal epithelium has high nerve density with relatively low inter-image parameter variability (32), but a sparse epithelial IC population. The use of images from healthy individuals in the present study was in recognition that the cornea has fewer epithelial ICs under physiological vs. inflammatory conditions. The image sample sizes determined in the present study are thus based on homeostatic corneal epithelial IC levels and provide a conservative estimate of the required sample size when relatively few ICs are present; the reported sample sizes are thus expected to remain robust when analyzing IVCM images with more ICs, such as diseased corneas. Although, this ideally should be confirmed in different disease states, acknowledging that corneal epithelial IC density may vary both in relation to absolute numbers and the region examined, dependent on the condition etiology. A further reason for the approach taken in the current paper is that healthy individuals often serve as controls in disease or intervention studies, and it is important to have reliable estimates in both participant populations.

In terms of image selection methods, a frequent approach involves an observer manually identifying a designated number of “representative images” for analysis; the use of three such images is common (9, 15, 19–22). However, observer bias might be expected to affect the validity of corneal epithelial IC density estimates derived from a small, subjectively curated image set (17). To consider this question, Kheirkhah et al. evaluated the mean corneal epithelial IC density calculated from “three representative images” selected by one observer with the value obtained from quantifying ICs in a wide-view composite image of the central cornea (covering 1.29 ± 0.64 mm^2^ of corneal area) (16). Although these authors reported no overall significant difference in the estimated values across the study population, they noted considerable differences between the methods as a function of cell density in individual participants (16). Furthermore, the average corneal area used to derive the benchmark value from the composite images was approximately half of that in the current study (and likely equivalent to about eight non-overlapping IVCM images). The present study focused on analyses at the participant (rather than study population) level, evaluating both the inter-observer consistency of the “three representative images” selection approach, and the level of precision relative to using three random IVCM images, in both central and peripheral cornea. These analyses identified poor inter-observer consistency for epithelial IC density estimates (as determined using the ICC), and a similar level of precision to three randomly selected images (as determined by assessing variance equality). These findings were conserved across the central and peripheral cornea. Together, these findings indicate that using “three representative images,” selected by experienced observers, to quantify corneal epithelial IC densities is likely to be inconsistent and suboptimal with respect to the level of sampling, and imprecise when compared to the “true mean”.

Considerations relevant to the interpretation of the current study include that, based on the prior work of Vagenas et al. (27), the “true mean” IC density has been taken as the average cell density calculated from 16 random, non-overlapping images, per participant, in each corneal region. The optimal image sample size was based on a pre-specified acceptable level of accuracy (27) for the estimated values of corneal epithelial IC density, which is an estimate within 30% of the true mean, 95% of the time. In the current study, only images of right eyes were acquired and analyzed, based on previous findings that corneal sub-basal nerve plexus parameters are highly correlated between eyes in an individual (33, 34). As such, we could not evaluate potential inter-eye asymmetries. We would expect, although could not identify direct evidence for, corneal epithelial IC densities also being similar between right and left eyes in healthy corneas (33, 34). Some indirect evidence for this relationship derives from research in unilateral corneal infection, where it has been shown that contralaterally clinically unaffected eyes show increased corneal epithelial IC densities; this was suggested to result from coordinated, bilateral interactions between the nervous and immune systems (35). Central corneal epithelial IC density measures similar to those reported in the present study have also been described in two recently published studies that analyzed a total of six images (i.e., three per eye) in healthy populations (36, 37).

We also acknowledge that the level of consistency between observers in the current study may not be generalizable, but instead represents the extent of agreement within this group of observers. All corneal epithelial ICs were quantified in the density calculations; morphological subtypes, which may represent either distinct cell populations or cells at different states of maturation, were not considered separately. It would be predicted that higher optimal image sample sizes may be required if distinct cell populations intend to be quantified. Some recent studies have assessed the infero-central corneal whorl region and noted that it is an area where round-shaped “globular” cells congregate (38–40). The current study determined optimal image sample sizes for deriving epithelial IC estimates in the central and peripheral corneal regions; the whorl region was not evaluated. This could be a topic for future research, noting that the corneal whorl will be inherently limited in its potential sampling area as it is a relatively small anatomical region of the cornea.

In conclusion, the present study finds that to minimize the likelihood of under-sampling at the participant level of a study, the average cell density value from quantifying 12 random, non-overlapping IVCM images (400 μm × 400 μm) should be used for corneal epithelial IC density estimates for the central cornea, and seven equivalent images should be used for the peripheral cornea. This study also finds that using “three representative images,” selected by experienced observers, to derive corneal epithelial IC density estimates from IVCM images has poor inter-observer consistency, and leads to imprecise estimates that are similar to random under-sampling.

## Data Availability Statement

The datasets presented in this article are not readily available because our current Ethics Approval does not permit data sharing, even on anonymised data. If there was as external request for access to the raw, anonymised data, the corresponding author could seek approval of an Ethics Amendment from the Ethics approval for this purpose. Requests to access the datasets should be directed to LD, ldownie@unimelb.edu.au.

## Ethics Statement

The studies involving human participants were reviewed and approved by the University of Melbourne Human Research Ethics Committee. The patients/participants provided their written informed consent to participate.

## Author Contributions

LD and HC conceived and designed the study and revised the manuscript. XZ collected the data. MW performed the data analyses. XZ and MW drafted the manuscript. All authors contributed to the interpretation of data, agreed to be accountable for all aspects of the work, and approved the final submitted version of the manuscript.

## Conflict of Interest

The authors declare that the research was conducted in the absence of any commercial or financial relationships that could be construed as a potential conflict of interest.

## Publisher’s Note

All claims expressed in this article are solely those of the authors and do not necessarily represent those of their affiliated organizations, or those of the publisher, the editors and the reviewers. Any product that may be evaluated in this article, or claim that may be made by its manufacturer, is not guaranteed or endorsed by the publisher.

## Footnotes

1
https://www.r-project.org/
